# Molecular mechanism of VE-cadherin in regulating endothelial cell behaviour during angiogenesis

**DOI:** 10.3389/fphys.2023.1234104

**Published:** 2023-08-02

**Authors:** Weijin Nan, Yuxi He, Shurong Wang, Yan Zhang

**Affiliations:** ^1^ Department of Ophthalmology, The Second Hospital of Jilin University, Changchun, China; ^2^ Department of Ophthalmology, China-Japan Union Hospital of Jilin University, Changchun, China; ^3^ Department of Ophthalmology, Shanghai General Hospital, Shanghai Jiao Tong University School of Medicine, Shanghai, China

**Keywords:** VE-cadherin, angiogenesis, endothelial cells, adhesion junctions, molecular mechanism

## Abstract

Vascular endothelial (VE)-cadherin, an endothelium-specific adhesion protein, is found in the junctions between endothelial cells (ECs). It’s crucial to maintain the homogeneity of ECs. Keeping and controlling the contact between ECs is essential. In addition to its adhesive function, VE-cadherin plays important roles in vascular development, permeability, and tumour angiogenesis. Signal transfer, cytoskeletal reconstruction, and contractile integrating, which are crucial for constructing and maintaining monolayer integrity as well as for repair and regeneration, are the foundation of endothelial cell (EC) junctional dynamics. The molecular basis of adhesion junctions (AJs), which are closely related and work with actin filaments, is provided by the VE-cadherin-catenin complex. They can activate intracellular signals that drive ECs to react or communicate structural changes to junctions. An increasing number of molecules, including the vascular endothelial growth factor receptor 2 (VEGFR2) and vascular endothelial protein tyrosine phosphatase (VE-PTP), have been connected to VE-cadherin in addition to the conventional VE-cadherin-catenin complex. This review demonstrates significant progress in our understanding of the molecular mechanisms that affect VE-cadherin’s function in the regulation of EC behaviour during angiogenesis. The knowledge of the molecular processes that control VE-cadherin’s role in the regulation of EC behaviour during angiogenesis has recently advanced, as shown in this review.

## Graphical Abstract

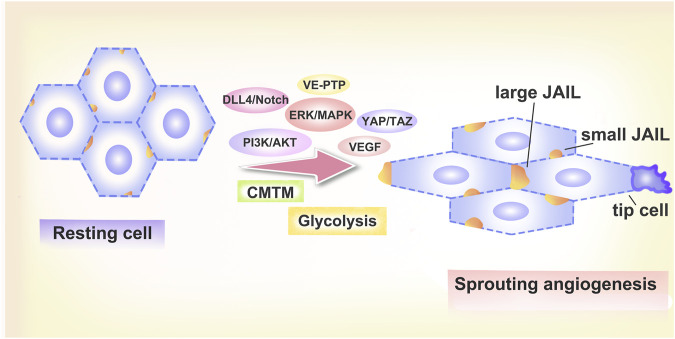

## 1 Introduction

Angiogenesis is a complicated process whereby already-existing blood vessels are exploited to create new ones (Potente et al., 2011). Angiogenesis is essential for normal body development, tumour growth, and migration (Hou et al., 2018). The endothelial cells (ECs) perform a formidable task in this process. ECs alter their behaviour in response to elements including proliferation, migration, and differentiation when exposed to various biomechanical signals from the environment (Vestweber et al., 2009). Angiogenesis is fueled by EC movement and rearrangements, which are essential for vascular development, including sprouting, migration, and lumen formation (Cao et al., 2017). For sprouting to begin, ECs must transform into tip cells and stalk cells with various morphologies and functional characteristics (Gerhardt et al., 2003). The tip cells of angiogenic sprouts are directly attached to stalk cells, and they are responsible for elongating the sprout and branching the vessel (Jakobsson et al., 2010). Adhesion junctions (AJs) based on vascular endothelial (VE)-cadherin connect ECs to one another. The development of angiogenic sprouts in vascular ECs requires remodelling of the AJs (Chrifi et al., 2019). Therefore, sprouting angiogenesis requires a stringent regulatory system that manages the development and termination of intercellular junctions between ECs.

Endothelial cell-cell adhesion, which is a vital part of preserving vascular integrity, is made up of VE-cadherin, commonly referred to as CDH5 (Giannotta et al., 2013). ECs express relatively high levels of VE-cadherin (Grimsley-Myers et al., 2020). Besides assisting endothelial adhesion, this protein is actively involved in regeneration, vasculogenesis, angiogenesis, inflammation, and tumour progression through VE-cadherin phosphorylation, endocytosis, and filamentous actin (F-actin) remodeling (Viallard and Larrivée, 2017). Similar to other cadherin superfamily members, the VE-cadherin structure is made up of a transmembrane structural domain, a cytoplasmic tail and an extracellular structural domain containing five calmodulin repeats (EC1-EC5) (Nollet et al., 2000). The extracellular domain EC1 has been demonstrated to be a target for antibodies, antibodies that target EC3–EC4 also have an impact on VE-cadherin adhesion and clustering. In addition, they affect vascular structure development, apoptosis, and endothelial cell permeability (Corada et al., 2001). Recent research have also revealed that Glc-82 lung cancer cells cultured in 3D matrigel were prevented from proliferating and forming capillary-like structures by antibodies to cadherin and binding of its epitope to the fourth extracellular domain (EC4). Inhibiting AKT phosphorylation served as a conduit for this action (Ding et al., 2018). These results underline how crucial it is to comprehend the structural underpinnings of cell adhesion mediated by VE-cadherin as a foundation for developing treatment strategies intended to particularly control angiogenesis and vascular permeability. Intercellular signals at intercellular contact sites can be transmitted to the cell interior by VE-cadherin via transmembrane and cytoplasmic binding partners (Ding et al., 2018). VE-cadherin, which is connected to p120-catenin and plakoglobin through its cytoplasmic domain, provides the “basic” organization of AJs (Grimsley-Myers et al., 2020) and through *α*-catenin to the cytoskeleton of F-actin, finally forming a VE-cadherin-catenin complex. The mutual reactions between VE-cadherin and its binding partners are adjusted depending on the condition of the endothelium, resulting in a suitable cellular response. However, it is yet unclear what molecular processes underlie the spatiotemporal cycling of AJs between ECs. In this review, we go over the molecular processes that influence the behaviour of ECs induced by the VE-cadherin-catenin complex during angiogenesis.

## 2 VE-cadherin promotes EC survival and proliferation during angiogenesis

**FIGURE 1:**
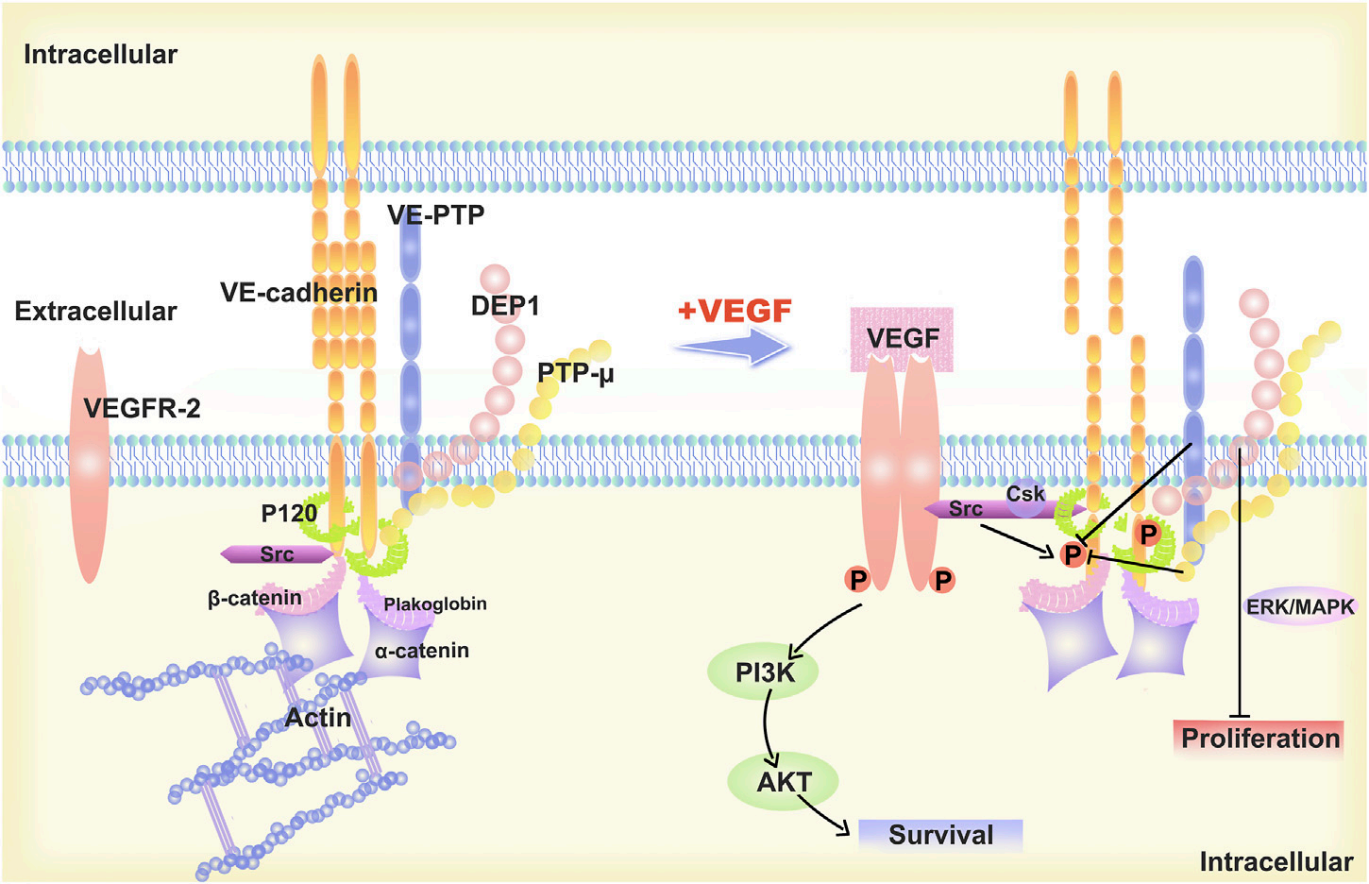
Description of the structural model of VE-cadherin and changes upon stimulation with VEGF-A. The “basic” organization of the AJs is provided by VE-cadherin, which is linked to p120-catenin and b-catenin, or platelet erythrocyte, through its cytoplasmic structural domain. The VE-cadherin is linked to p120-catenin and b-catenin or platelet haemoglobin via its cytoplasmic structural domain and reaches the cytoskeleton F-actin via alpha-catenin, ultimately forming the VE-cadherin-catenin complex. In resting endothelial cells, VE-cadherin aggregates at the AJs and is maintained non-phosphorylated by phosphatases such as VE-PTP and PTP-A. When stimulated by VEGF-A, VEGF R2 binds to VE-Cadherin and Src and the AJs are disrupted. VEGFR2 and Src kinase activity leads to VE-cadherin phosphorylation, which induces cell survival through the PI3K/AKT signaling pathway, cell proliferation via ERK/MAPK signaling pathway, and cell migration and differentiation.

Differences in VE-cadherin activity reflect the diverse behaviours of ECs during vascular quiescence and angiogenesis (Etienne-Manneville, 2020). In proliferating cells, the function and signaling of VE-cadherin is profoundly altered, and that this transition is accompanied by increased gene transcription and phosphorylation of proteins with tyrosine residues (Muramatsu et al., 2017). It has been shown that VE-cadherin-deficient ECs are round and less mobile (Feraud et al., 2001). In resting cells, VE-cadherin molecules assemble at AJs in the absence of vascular endothelial growth factor (VEGF). Vascular endothelial protein tyrosine phosphatase (VE-PTP) and PTP-A are phosphatases that sustain dephosphorylation (Huo et al., 2019). When VEGF is present, VEGFR2 binds to VE-cadherin and Src, which disrupts the AJs. The tyrosine phosphorylation of VE-cadherin is caused by the action of VEGFR2 and Src kinase (Liu et al., 2020). The VEGFR2/VE-cadherin combination induces cell survival through the (phosphatidylinositol 3 kinase) PI3K/(protein kinase B) AKT signaling pathway (Zhang et al., 2022), the (extracellular regulated protein kinases) ERK/(mitogen-activated protein kinase) MAPK pathway activation stimulates cell proliferation (Biagioni et al., 2021), while CDC42 activation induces membrane protrusion (Martin et al., 2016). (Figure 1)

## 3 VE-cadherin promotes EC migration and vascular lumen formation during angiogenesis

Angiogenesis is a complex process that involves several steps. It is currently unclear how the local microenvironment selectively acts on ECs to cause capillaries to grow in an orderly manner through sprouting and intussusception. Vascular sprouting, however, also needs AJs disruption. Junctional strength is regulated by VE-cadherin endocytosis, and vascular stability is decreased by a reduction in surface VE-cadherin levels. When sprouting occurs, functional EC rearrangement is driven by VE-cadherin junction dynamics (Bentley et al., 2014). Remodeling of VE-cadherin-based AJs is necessary for ECs movement and rearrangement in angiogenic sprouts (Bentley et al., 2014). VE-cadherin promotes EC migration and vascular lumen formation by enhancing the connections between ECs.

### 3.1 VEGF-VEGFR signaling

The differentiation of ECs into tip cells and stalk cells with unique morphologies and functional characteristics is necessary for sprout initiation. ECs stimulated with VEGF adopt a promising phenotype. Tip cells are the ECs that are at the head of the developing capillaries (Gerhardt et al., 2003). Tip cells react to extracellular matrix, growth factors, and other stimuli that are alluring or repellent. They are motile, invasive, and extend many filopodia. While succeeding ECs (stalk cells) multiply and help to maintain the structural and functional integrity of developing vessels, a small percentage of ECs transition to the tip cell phenotype and start sprouting during angiogenesis. Additionally, AJs need to be broken during angiogenesis sprouting for EC movement and morphological adaptation into tip cells and stalk cells. This is made possible by a reduction in the adhesive strength (Chrifi et al., 2019). An essential component of endothelial AJs is VE-cadherin. There is strong evidence to support the idea that in the process of angiogenesis, VE-cadherin plays a crucial part.

VEGF is one of the initiators of angiogenic factors, which have a very strong stimulatory effect on the proliferation, migration, and chemotaxis of the vascular endothelium in various organs and even the whole body (Ferrara, 2004). The VEGF family is comprised of several members that perform various functions in humans. Three distinct tyrosine kinase receptors are capable of interacting with VEGF ligands: the 1–3 VEGF receptors (also known as Flt1, Flk1, and Flt4) (Olsson et al., 2006). A VEGF-A gradient frequently kickstarts angiogenesis by activating tip cells derived from endothelium, which spread filopodia and produce sprouts when they expand into tissues that express VEGF (Gerhardt et al., 2003). It is believed that stalk cells and tip cells contend for the position of the tip cells through dynamic transformation (Jakobsson et al., 2010). Angiogenic ECs need to communicate with cell junctions in a manner that is precisely coordinated and heavily rely on the dynamics of VE-cadherin in order for them to move and change their position (Bentley et al., 2014; Cruys et al., 2016).

It is widely recognised that, in VEGF-activated ECs, the reduction in junctional integrity is mainly due to the reduced presence of VE-cadherin in AJs. It has been demonstrated that VEGF stimulates the endocytosis of VE-cadherin via controlling (guanine nucleotide exchange factor) Vav2 via Src (Spring et al., 2012). When VEGFA is activated, the VEGFR2 dimer is created. As a result, the SRC phosphorylates VAV2, resulting in the activation of Rac, a small GTPase. Activated Rac induces P21-activated kinase (PAK) to mediate phosphorylation of conserved serine residues (S665) in the cytoplasmic tail of VE-cadherin cells, leading to recruitment of β-arrestin2. This pathway activates the endocytosis of statin-dependent VE-cadherin, thereby reducing intercellular adhesion (Gavard and Gutkind, 2006). In specifically, the derivable passivation of the cadherin5 (Cdh5) gene, which expresses VE-cadherin, demonstrates that angiogenic sprouting is increased *in vitro* and *in vivo* when VE-cadherin expression is somewhat decreased (Gaengel et al., 2012; Polet and Feron, 2013). Together, our findings show that VE-cadherin equilibrium at intercellular junctions is essential for the initiation and development of angiogenesis. Moreover, VE-cadherin suppresses VEGFR2-Rac1-dependent angiogenesis (Wang et al., 2021). The VE-cadherin signaling network is intricate and varies in the developing or dormant vasculature as well as under various functional situations.

### 3.2 Integrins

Integrins primarily mediate cell-to-cell and cell-to-extracellular matrix adhesion. It acts as a receptor for matrix molecules, receives signals from extracellular molecules and transduces them through signaling pathways to influence gene expression, thereby regulating processes such as cell proliferation, cell cycle and cell migration (Kechagia et al., 2019). In angiogenesis, integrins participate in lumen formation and vascular network construction by regulating the adhesion and migration of ECs. By studying the integrin β1-subunit in the mouse retina, the researchers found that β1 integrin promotes endothelial cell sprouting but have a negative regulatory effect on proliferation (Yamamoto et al., 2015). And it was found that deletion of integrin β1 impaired VE-calcineurin distribution and p120 binding (Yamamoto et al., 2015). Previous studies have shown that E-cadherin can control integrin activity (Valat et al., 2022) and is associated with the small GTPase Rap1 (Shah et al., 2019), which also promotes cell junction formation and may mediate bidirectional cross-talk between cadherins and integrins in ECs. However, given the enormous complexity of the signaling interactions, the synergistic interaction between integrins and VE-cadherin remains to be further investigated.

**FIGURE 2:**
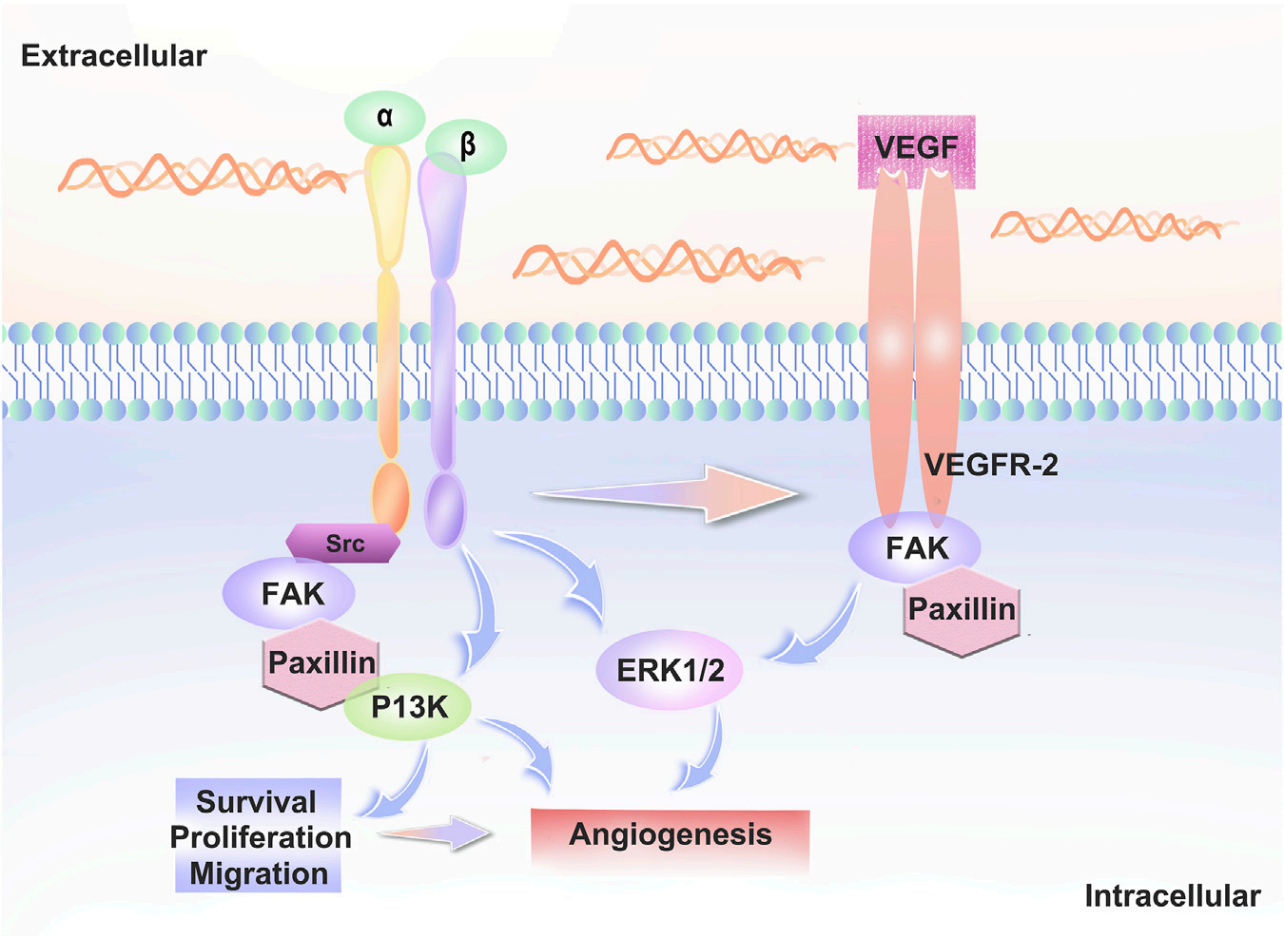
Interaction between integrins and VEGFR2. When VEGF-A binds to VEGFR2, intracellular signaling pathways such as PI3K, ERK and FAK are activated. Src family kinase (SFK) interacts with the vascular endothelial growth factor receptor to regulate vascular growth factor gene expression, regulates cell proliferation via the MAPK-ERK pathway and interacts with integrins to regulate cell adhesion and migration.

VEGFR2 signaling is regulated by several receptors, including integrins. Signaling crosstalk between integrins and VEGFR2 is necessary for a fully realized angiogenic program (Bazzazi et al., 2018). Integrins α5β1 and αvβ3 play a particularly important role in angiogenesis (Lakshmikanthan et al., 2011; Hu et al., 2019).Rap1b promotes vegf-mediated angiogenesis by promoting the activation of VEGFR2 in ECs via integrin αvβ3 (Lakshmikanthan et al., 2011). When VEGFA binds to VEGFR2, many intracellular signaling pathways are activated and interact with integrins to regulate cell adhesion and migration. Thus, integrins can directly or indirectly influence the distribution and function of VE-cadherin and thus lead to changes in endothelial cell behaviour. (Figure 2).

### 3.3 Dll4-notch signaling

During sprouting angiogenesis, ECs actively compete for the location of tip cells, and (Neurogenic locus notch homolog protein) Notch signaling is associated with the dynamic rearrangement of ECs (Jakobsson et al., 2010). Specialized endothelial tip cells facilitate blood vessel sprouting toward VEGF-A gradients in sprouting angiogenesis (Gerhardt et al., 2003). Delta-like-4 (Dll4), which is ligand of Notch that binds to Notch receptors expressed on surrounding cells to inhibit the amount of sprouting cells, is stimulated by high VEGF-A signaling. When nearby cells’ Notch signaling is active, it lessens those cells’ receptivity to VEGF-A (Lagendijk and Hogan, 2015). Thus, VEGF-A signaling levels are high in tip cells and low in stalk cells due to the activation of DLL4-Notch signaling (Arima et al., 2011).

**FIGURE 3:**
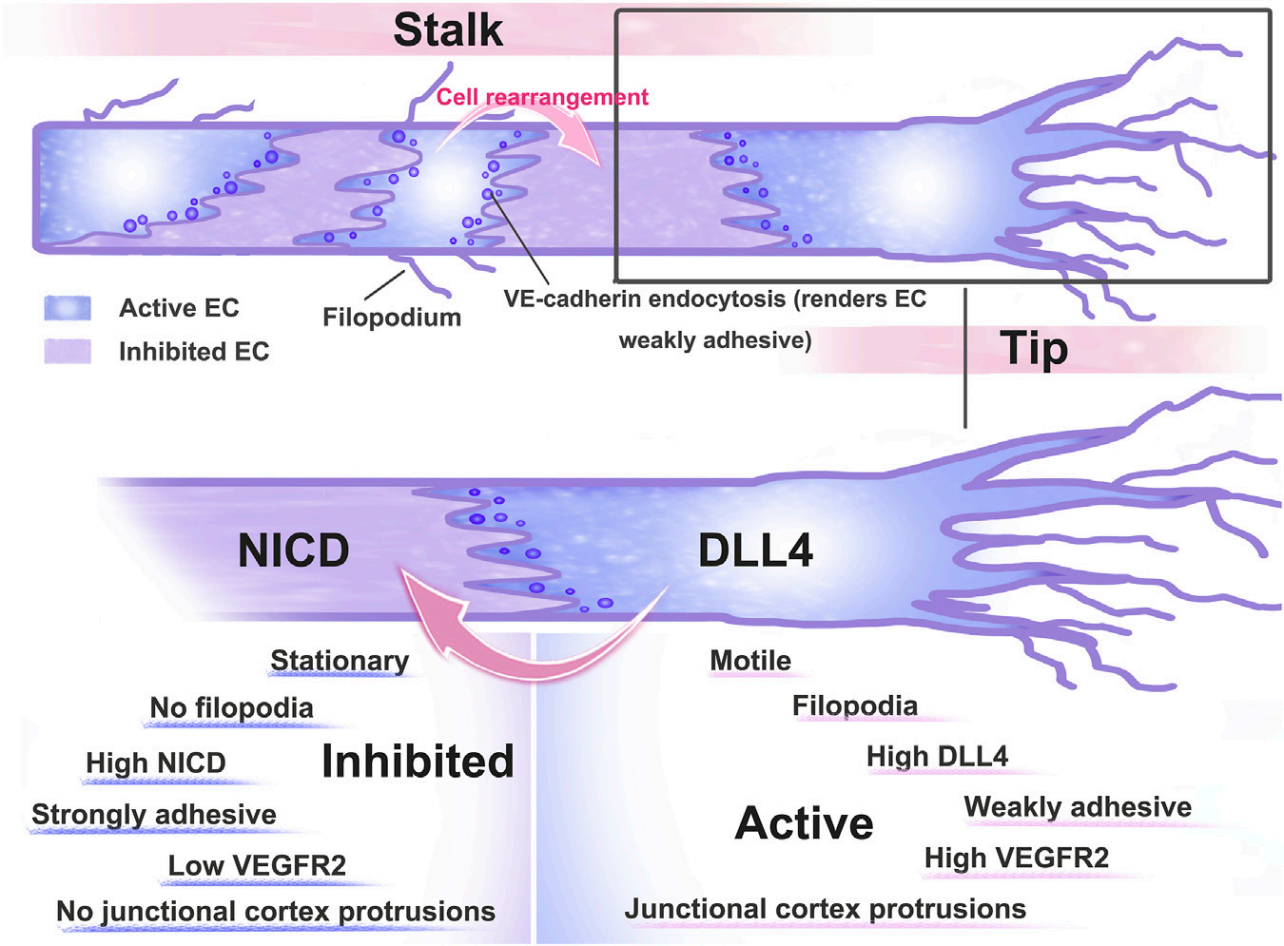
Pattern diagram of dynamic rearrangement of ECs. After ECs receive VEGF-A stimulation, AJs are disrupted and ECs differentiate into tip and stem cells, with tip cells extending towards the filopodium and directing sprout as they extend towards VEGF-expressing tissues. Tip stalk cells are thought to be dynamically transformed as they compete for the position of tip cells. To limit the number of angiogenesis sprouting cells, high VEGF signaling activates the expression of the Notch ligand Dll4, which binds to the Notch receptor expressed on neighbouring cells. Activation of Notch signaling in neighbouring cells reduced their responsiveness to VEGF.

Bentley et al. pointed out that the reduction in Notch signaling *in vivo* and *in vitro* showed that Notch is key for maintaining junctional heterogeneity (Bentley et al., 2014). Less active junctions are produced when the pathway is overactivated using a compositively active version of the Notch1 intracellular domain (NICD). In cells that experience high Notch activation, the junctional VE-cadherin-GFP protein turnover is decreased. These findings imply that failure of VE-cadherin’s differential linking activity, which frequently results in heterogeneity in cell behaviour, may be to blame when VEGF or Notch levels are disturbed (Bentley et al., 2014). According to research, one signal that facilitates Notch1 in ECs and triggers Rac1-mediated cortical actin assembly, which strengthens VE-cadherin at intercellular junctions in a transcriptionally independent way, is shear stress (Polacheck et al., 2017). The DLL4-Notch signaling pathway is involved in several stages of angiogenesis and is interlaced with other cytokines or signaling pathways to complete the complex regulation of angiogenesis. Heterogeneity is essential to angiogenesis. Both VE-cadherin and Notch signaling play crucial roles in decisive events. (Figure 3)

### 3.4 VE-PTP

The endothelial Tie-2 receptor tyrosine kinase inhibitor vascular endothelial protein tyrosine phosphatase (VE-PTP) interacts to VE-cadherin via its proximal structural region that resembles fibronectin (Nawroth et al., 2002). Tyrosine residues are phosphorylated and dephosphorylated to achieve VE-cadherin’s adhesion (Nawroth et al., 2002). VE- PTP stabilizes VE-cadherin adhesion, causing VE-cadherin to stabilize VE-cadherin adhesiveness by dephosphorylating it at Tyr658 and Tyr685 (Siragusa et al., 2021). Leukocyte extravasation and endothelial cell permeability are enhanced by VE-PTP knockdown, and blocking the separation of VE-PTP and VE-cadherin reduces these occurrences (Broermann et al., 2011). Insufficient VE-PTP impairs cell polarity and lumen development in mouse embryoid bodies through enhancing the activity of VEGFR2 in stalk cells and tyrosine phosphorylating VE-cadherin (Hayashi et al., 2013). Reduced VE-cadherin phosphorylation and junctional activity of VEGFR2 are both regulated by VE-PTP in a Tie2-dependent manner (Hayashi et al., 2013). Studies have shown that VE-PTP can stabilize the VE-cadherin junction and endothelial barrier through manner that is independent of phosphatase. Several researchers found that through the reduction of VE-cadherin internalization rate, VE-PTP stabilized VE-cadherin junctions (Juettner et al., 2019). By directly interacting with GEF-H1 and blocking its ability to bind to RhoA, VE-PTP carries out a crucial junctional role by lowering RhoA activity and tension at the VE-cadherin junction (Juettner et al., 2019). This is a crucial element controlling how stable AJs are in vessels. These investigations lay a theoretical foundation for the interaction between VE-PTP and VE-cadherin at the molecular level and highlight the special function of VE-cadherin in the behaviour of ECs during angiogenesis.

### 3.5 YAP and TAZ

Yes-associated protein 1 (YAP) and transcription regulator 1 (TAZ), effectors of the Hippo signaling pathway, control proliferation and apoptosis and are crucial for organ development (Piccolo et al., 2014). Several researchers have created animal models with endothelial-specific loss- and gain-of-function to study the roles of YAP and TAZ in the vasculature. They found that TAZ and YAP were both functionally expressed, essential for sprouting angiogenesis in ECs, and both were expressed. YAP and TAZ deficiency reduced the frequency of junction-associated intermediate lamellipodia and VE-cadherin turnover (Neto et al., 2018). Thus, YAP/TAZ balances endothelial cell rearrangement in angiogenesis, enhances VE-Cadherin turnover, stimulates cell migration, and maintains barrier function. But BMP signals help to some extent in doing this. Sprouting angiogenesis is essential for the establishment of blood vessel networks. It is still unclear how ECs form sufficient numbers and a suitable arrangement to form functioning circulatory networks.

YAP is also a mechanotransduction protein that constantly shuttles between the nucleus and cytoplasm, with nuclear localization being a key determinant of its activation to regulate downstream target genes.YAP/TAZ delivers mechanotransduction cues from the extracellular matrix to control cell survival mediated through Rho and the cytoskeleton (Dupont et al., 2011). ARHGAP18 was identified as a downstream effector of YAP, which regulates cell shape and size. Depletion of ARHGAP18 leads to disruption of junctions, such as loss of localization of VE-Cadherin in these regions and concomitant localization of YAP to the nucleus (Coleman et al., 2020). Therefore the YAP/ARHGAP18 axis is part of the mechanical transduction cascade in ECs in response to shear stress. At the same time, VE-cadherin complexes act as endothelial force sensors, and mechanical transduction in endothelial cells triggers local cytoskeletal remodeling and also activates global signals that alter peripheral cell-cell connections and disrupt cell-cell contacts far from the site of force application (Barry et al., 2015).

### 3.6 CMTM

A member of the (CKLF-like MARVEL transmembrane domain) CMTM gene family is CMTM1-8. This gene family’s products have a significant impact on the immune system, the male reproductive system, and tumor formation (Wu et al., 2020). At baseline and in response to VEGFA stimulation, CMTM3 expression encourages VE-cadherin internalization, but CMTM3 knockdown prevents this action. A crucial part in angiogenesis is played by CMTM3 (Chrifi et al., 2017). Since the instability of intercellular connections encourages EC mobility and cell differentiation, the weakening of intercellular junctions is essential for the early stage of angiogenic sprouting. According to this study, CMTM3 significantly boosted VE-cadherin internalization through the medium of VEGFA, which in turn weakened the AJs between cells (Chrifi et al., 2017). CMTM3 regulates the pool of cell surfaces and participates in VE-cadherin turnover. Therefore, CMTM3 regulates cell-cell adhesion at AJs and helps to prevent vascular sprouting. To pinpoint the particular mechanism by which CMTM3 promotes VE-cadherin internalization, more investigation is required. Additionally, CMTM4 improves VE-cadherin internalization (Chrifi et al., 2019). Loss of CMTM4 significantly reduces vascular sprouting, as demonstrated in zebrafish larvae and *in vitro* in a 3D vascular experiment (Chrifi et al., 2019). Similar to CMTM3, CMTM4’s ability to promote angiogenesis is influenced by the control of VE-cadherin on the cell surface. CMTM4 overexpression promotes VE-cadherin internalization at basal levels and in reaction to VEGFA stimulation. By encouraging the cell membrane recirculation of VE-cadherin to endothelial AJs, CMTM4 controls angiogenesis.

### 3.7 PACSIN2/EHD4/MICAL-L1

VE-cadherin binds to the actin cytoskeleton intracellularly via the *α*-catenin and β-catenin proteins. Actomyosin’s cytoskeleton or external forces of mechanics are in charge of controlling this binding (Takeichi, 2014; Dorland and Huveneers, 2017). Often, collective control of cell-cell interactions in tissues occurs through polarized cell-cell junction dynamics (Takeichi, 2014). Asymmetric AJs are produced as a result of the stress that these driving pressures at the border between the leading and following cells place on cell-cell connections during sprouting angiogenesis (Malinova and Huveneers, 2018). The local curvature of the junctional plasma membrane and the curvature-sensing F-BAR protein both assist in the development of asymmetric tensile AJs. Follower cells exclusively have (protein kinase C and casein kinase 2 substrate in neurons type 2) PACSIN2 towards the rear of asymmetric AJs (Dorland et al., 2016). Whenever pacsin2’s functionality is hampered, there are higher amounts of internalized VE-cadherin (Dorland et al., 2016). They observed that the F-BAR protein PACSIN2, which functions as an indicator between the leader and follower cells (Malinova et al., 2021). VE-cadherin local trafficking is regulated by the junctional PACSIN2/(EH Domain Containing 4) EHD4/(MICAL Like 1) MICAL-L1 complex, which also regulates polarized endothelial movement and angiogenesis.

### 3.8 JAIL

When the vascular ECs in development divide into tip and stalk cells, they can interact and transform into one another. Stalk cells can move both forward and backward during interconversion in the current vascular-like system, which results in a shift in cell polarity and individually controlled connections. A structure that shows that local decrease of VE-cadherin at particular endothelial junction locations starts the production of actin-driven plasma membrane protrusions was recently reported in cell culture research. (Junction-associated intermittent lamellipodia) JAIL was the name given to these structures (Polet and Feron, 2013). E-cadherin and actin dynamics are related in migrating ECs as a result of JAIL’s subsequent generation of extra VE-cadherin adhesion sites and the interdependent driving of VE-cadherin and actin dynamics. In angiogenesis models and in actual beings, the distribution of VE-cadherin was polarized with cell elongation. At the conduction cell pole, directional cell migration takes place. These cells typically exhibit altered VE-cadherin patterns, which leads to the creation of large JAILs. Extensive VE-cadherin plaques that can serve as adhesion anchors and permit cell migration are produced as a result of JAIL formation (Cao et al., 2017). The expanding lateral connections reveal a faint linear VE-cadherin pattern with mild JAIL, leading cells to move in proximity to one another (Cao et al., 2017). JAIL and VE cadherin plaques alternate in an interrupted pattern at the cell poles. The recurring cycle between damaged VE-cadherin patterns and JAIL-mediated generation of new VE-cadherin adhesion sites is essential for EC rearrangement during angiogenesis.

### 3.9 Mechanosensory complex

Mechanical forces control EC survival, mobility, proliferation, and (extracellular matrix) ECM modification, all of which are essential for angiogenesis, according to studies (Li et al., 2005). In vascular biology, the dynamic response of cells to mechanical pressures is crucial. Through AJs, ECs attach mechanically to nearby cells. Within AJs, VE-cadherin, VEGFR2/3, and (platelet endothelial cell adhesion molecule 1) PECAM1 form a mechanosensory complex (Kutys and Chen, 2016). Within this complex, VE-cadherin functions as an adaptor, PECAM1 transmits mechanical force directly, and VEGFR2 encourages biochemical signaling (Hahn and Schwartz, 2009). Through its transmembrane domain, VE-cadherin contributes in the interaction between PECAM1 and VEGFR2 to promote decreased VEGFR activity in response to mechanical stimulation of PECAM1 (Kutys and Chen, 2016). In response to increasing matrix stiffness, VE-cadherin-mediated cell-cell junctions failed to form in both *in vitro* and *ex vivo* environments, leading to the rupture of barrier integrity (Huynh et al., 2011). Angiogenesis in tumors has also been shown to be disrupted in this manner. Angiogenesis is aided by cellular link breakdown. There have been several alleged mechanical force sensors and transducers discovered, but they need to be verified by more research.

### 3.10 Glycolysis and VE-cadherin

The main method used by ECs to produce ATP is glycolysis (Polet and Feron, 2013). Endothelial tip cells’ glycolysis rises during sprouting angiogenesis. Silencing the glycolytic activator (P6-phosphofructo-2-kinase/fructose-2,6-biphosphatase 3) FKFB3 reduced angiogenic sprouting, whereas inhibition or stimulation of respiration had no effect (De Bock et al., 2013; Liu et al., 2022). The pyruvate kinase (pyruvate kinase) PKM2 is required for both *in vitro* and *in vivo* sprouting angiogenesis, according to Gomez-Escudero et al. (Gómez-Escudero et al., 2019). When HUVEC were stimulated with VEGFA and PKM2 was inhibited, VEGFA-induced VE-cadherin internalisation was significantly reduced in ECs due to PKM2 silencing compared to that in controls. PKM2 deficiency or suppression results in lower ATP levels close to endothelial junctions, which inhibits junction remodeling, group movement, and angiogenic sprouting. Additionally, JAlL production and VE-cadherin clathrin-mediated internalization are hampered (Gómez-Escudero et al., 2019). The balance between stable and unstable junctions was disturbed by PKM2 deletion, which decreased junctional VE-cadherin levels and increased the frequency of unstable junctions. This promotes the rearrangement of ECs and provides conditions for the sprouting of blood vessels.

## 4 VE-cadherin promotes vascular fusion

Circulatory fusion is required for the development of a closed circulatory system, but little is understood about the processes governing this important process, including cellular and molecular ones. In order to enhance vascular fusion, intermedin (IMD) has been identified to trigger VE-cadherin mobility and govern the dynamic dissociation and remodelling process of the VE-cadherin complex (Kong et al., 2020). To be more precise, it causes VE-cadherin to enter a state of continuous phosphorylation, which causes the dissociation of pre-existing VE-cadherin complexes and the synthesis of new VE-cadherin complexes, achieving a dynamic equilibrium between the dissociation/remodelling of VE-cadherin complexes. In addition, it promotes the anastomosis of adjacent blood vessels to continuously expand them, which occurs through connecting the lumens of the two sides of the blood vessels. This study elucidated the key molecular mechanisms that regulate vascular fusion, indicating a critical role of VE-cadherin.

## 5 Vasculogenic mimicry

Vasculogenic mimicry (VM) is the ability of cancer cells to arrange themselves into vascular-like structures for the goal of acquiring nourishment and oxygen without the aid of conventional blood vessels or angiogenesis (Fernández-Cortés et al., 2019). Over the past few decades, VE-cadherin has been identified in a wide range of tumor forms, and it is essential for the VM and aggressiveness of malignancies. Only extremely aggressive melanoma cells express VE-cadherin, which is absent from less aggressive tumor cells (Hendrix et al., 2001). The ability of aggressive melanoma cells to form angiogenic networks is compromised by the downregulation of VE-cadherin expression (Hendrix et al., 2001). However, it is currently unknown how VE-cadherin functions in aggressive tumor cells during VM. (Focal adhesion kinase) FAK, which phosphorylates VE-cadherin at Y658 in tumor-associated ECs, has recently been found to be crucial for modulating EC barrier function and tumor metastasis (Jean et al., 2014). VEGFR2 and integrin cooperate to activate the cytoplasmic tyrosine kinase FAK (Chen et al., 2012). These studies show that FAK acts as a key signaling switch in ECs that regulates AJ dynamics. The VE-cadherin phosphatase, or VE-PTP, was created by human malignant melanoma cells, and it combines with VE-cadherin and p120-catenin to form a complex that protects VE-cadherin against degradation by autophagy (Delgado-Bellido et al., 2020). There could be a connection between VM and VE-cadherin given their respective roles in angiogenesis. Future research should concentrate on controlling VE-cadherin phosphorylation.

## 6 VE-cadherin-mediated epigenetic mechanisms

The precise regulation of gene expression is fundamental to normal cell development and function. The mechanisms of angiogenesis remain largely unknown. Vascular endothelial cells have been extensively studied for triggering a series of signaling pathways involved in endothelial differentiation and vascular stabilisation, but little is known about VE-cadherin and its associated epigenetic regulatory mechanisms. Gene regulation occurs at multiple levels, including DNA-binding transcription factors, chromatin and histone post-translational modifications (Nacev et al., 2020). Polycomb group proteins, which regulate gene expression at the chromatin level, are integral to developmental processes (Zhao and Wu, 2021). Polycomb proteins typically assemble into two protein complexes, Polycomb repressive complex 1 (PRC1) and PRC2, that block gene expression through a variety of mechanisms, such as chromatin compaction, inhibition of transcription initiation or elongation, recruitment of transcriptional inhibitors and blocking binding of key activators (Di Croce and Helin, 2013).

The expression and aggregation of vascular endothelial cells upregulate the endothelial specific genes of claudin-5, VE-PTP and VWF, which play a key role in vascular stability, by inhibiting the activity of polycomb on the promoters of specific genes (Morini et al., 2018). The VE-cadherin/β-catenin complex also isolates the core subunit of PRC2 (Ezh2) on the cell membrane, preventing its nuclear translocation. Inhibition of Ezh2/VE-cadherin binding increases recruitment of claudin-5, VE-PTP, and VWF promoters by Ezh2, leading to gene downregulation that regulates EC differentiation and vascular maturation (Morini et al., 2018). These findings extend the understanding of Polycomb-mediated gene expression regulation in endothelial cell differentiation and vascular maturation. New therapeutic opportunities are opened through known mechanisms using pharmacological inhibition of Polycomb-mediated repressive systems to regulate endothelial gene expression and induce vascular normalization. In a mouse model of breast cancer, tumor cell enhancer of Ezh2-driven cytokines disrupts endothelial connections, and VE-cadherin expression is reduced, which can be saved in the presence of Salvianolic acid B (SalB) (Qian et al., 2022). SalB provides a major pathway for vascular normalization by regulating the interaction between tumor cells and ECs to restore vascular integrity. It provides more evidence for VE-Cadherin-mediated epigenetic regulation and a new idea for the treatment of angiogenesis.

## 7 VE-cadherin and vascular disease

VE-cadherin contributes to important and distinctive functional characteristics of ECs during angiogenesis. In particular, VE-cadherin interacts with β-catenin/α-catenin proteins to connect to the actin cytoskeleton (Dorland and Huveneers, 2017). The cytoskeleton has many important physiological functions, such as maintaining cell morphology, carrying out various cell movements, and transporting intracellular substances (Hohmann and Dehghani, 2019). F-actin is a basic cytoskeleton protein that maintains cell morphology and polarity. The rearrangement of F-actin is the molecular basis for increased endothelial permeability caused by inflammation and vascular injury (Seetharaman and Etienne-Manneville, 2020). Cell-cell adhesion and F-actin are tightly connected, and changes in F-actin function can affect both cell shape and cell-cell adhesion. Changes in cell-cell adhesion can cause F-actin to reorganize concurrently by triggering intracellular signal transduction pathways (Cerutti and Ridley, 2017).

Atherosclerosis, pulmonary hypertension, and cardiac fibrosis are vascular disorders that are impacted by the recombination of vascular structures. The precise regulatory mechanism of vascular remodelling during the formation of neointima and the generation of neointimal cells is yet unknown. Understanding the neointimal cell genesis and the molecular processes that control their development is essential for our comprehension of vascular disorders. The preservation of endothelial cell connections during (endothelial-to-mesenchymal transition) EndMT is facilitated by EC-CD45 expression in ECs, as demonstrated by research using an EC-specific lineage tracking system (VE-cadherin-BAC-Cre ERT2 mice) (Yamashiro et al., 2022). Additionally, ECs were discovered to be the source of neointima, offering fresh proof for the long-debated origin of neointima cells following vascular damage. It also provides a potential new treatment strategy for controlling neointima formation and interfering with angiogenesis.

To allow maternal blood to access the placenta during the development of the mammalian placenta, trophoblasts penetrate the meconium of the mother and modify the spiral arteries (Burton and Jauniaux, 2018). Endovascular invasion is the term for this process, which is assumed to be connected to the usefulness of trophoblast-absorbing vascular ECs. Invading trophoblast cells also express VE-cadherin, which serves as the primary molecular mechanism for the endovascular invasion of these cells (Sung et al., 2022). VE-cadherin deficiency was found to lead to failure of spiral artery remodeling, which results in reduced maternal blood flow to the placenta, causing fetal growth restriction and death (Sung et al., 2022). It implies that VE-cadherin deficit in trophoblast cells may be a significant contributor to the pathophysiology of preeclampsia. The fetal contribution to preeclampsia may be studied using a model with VE-cadherin deletion that is unique to trophoblasts.

Some retinal diseases, particularly diabetic retinopathy and age-related macular degeneration, are characterized by a breakdown in the structure and function of blood vessels, leading to abnormal vascular hyperplasia. Angiopoietin dysregulation is associated with diabetic retinopathy. Exposure of human retinal endothelial cells to hyperglycemia *in vitro* induces a significant increase in Ang2 mRNA and protein, which in turn stimulates the phosphorylation and degradation of VE-cadherin (Collazos-Alemán et al., 2022), thereby increasing vascular permeability and leading to macular edema.

Sepsis is a dysregulated immune response to infection that results in multiple organ dysfunction and death (Singer et al., 2016). Breakdown of EC barrier connections plays a major role in multiple organ damage and mortality in sepsis. High serum lactate levels have been recognized as a key biomarker of sepsis prognosis (Singer et al., 2016). The destruction of VE-cadherin is sufficient to induce disassembly of blood vessel walls and increase vascular permeability, leading to tissue edema (Yu et al., 2019). During sepsis, multiple cell-derived mediators, such as pro-inflammatory cytokines and injury-associated molecular patterns, impair the vascular barrier by inducing VE-cadherin endocytosis and degradation. The mechanism involves lactate phosphorylation by extracellular signal-regulated kinase 1/2 (ERK1/2) induced by G protein-coupled receptor 81 (GPR81) signaling. ERK1/2 phosphorylation promotes the activation of calpsin 1/2 to the proteolytic cleavage of VE-cadherin, followed by caveolin1/clathrin-1 mediated endocytosis of VE-cadherin (Yang et al., 2022). It provides a new way to study the metabolic crosstalk between lactate and endothelium.

## 8 Conclusion and future directions

Therefore, VE-cadherin-centered AJs are important for angiogenesis. A crucial component of the AJs connecting vascular ECs is VE-cadherin, whose structural and functional defects can cause AJs to break down. The regulatory mechanisms are unclear, although a range of pathogenic stimuli acting on ECs can result in aberrant activation and damage, instability of VE-cadherin and endocytosis (Grimsley-Myers et al., 2020), a reduction in VE-cadherin, weakening of AJs, and the beginning of EC differentiation. Furthermore, it is yet unclear how various pathogenic conditions affect the structure and functionality of VE-cadherin to cause the disruption of EC AJs. We concentrated on the molecular mechanisms that the VE-cadherin complex uses to control EC behaviour during angiogenesis. The protein VE-cadherin may bind to both external and intracellular protein-binding partners to form functional complexes that can either activate intracellular signals or affect the structure of junctions. The influence of several influencing variables on the VE-cadherin complex is now the subject of more investigation. However, further research is needed to determine the specific function and timing of VE-cadherin’s modulation of endothelial cell behavior. Further research is also necessary to determine the precise mechanism by which VE-cadherin influences cell rearrangement and vascular remodelling. In-depth research on the VE-cadherin-catenin-F-actin complex’s phosphorylation, endocytosis, and remodeling processes might help identify novel therapeutic targets for preventing angiogenesis and open up new therapeutic vistas. In addition, the regulation of gene expression by epigenetic mechanisms of VE-cadherin is a hot area, which involves potential new therapeutic opportunities to regulate endothelial gene expression and induce vascular normalization, which we need to continue to explore.

## Figures and Tables

**Table:** 

**ECs**	Endothelial cells
**VE-cadherin**	Vascular endothelial-cadherin
**VEGF**	Vascular endothelial growth factor
**VEGFR2**	Vascular endothelial growth factor receptor 2
**VE-PTP**	Vascular endothelial protein tyrosine phosphatase
**DLL4**	Delta-like protein 4
**Notch**	Neurogenic locus notch homolog protein
**AJs**	Adherens junctions
**F-actin**	Filamentous actin
**AKT**	Protein kinase B
**MAPK**	Mitogen-activated protein kinase
**ERK**	Extracellular regulated protein kinases
**PI3K**	Phosphatidylinositol 3 kinase
**Vav2**	Guanine nucleotide exchange factor
**PAK**	p21-activated kinase
**YAP**	Yes-associated protein 1
**TAZ**	Transcription regulator 1
**CMTM family**	CKLF-like MARVEL transmembrane domain containing family of genes
**PACSIN2**	Protein kinase C and casein kinase substrate in neurons 2 Gene
**EHD4**	EH Domain Containing 4
**MICAL-L1**	MICAL Like 1
**JAIL**	Junction-associated intermittent lamellipodia
**PECAM1**	Platelet endothelial cell adhesion molecule 1
**ECM**	Extracellular matrix
**PFKFB3**	6-phosphofructo-2-kinase/fructose-2,6-biphosphatase 3
**PK**	Pyruvate kinase
**IMD**	Intermedin
**VM**	Vascularogenic mimicry
**FAK**	Focal adhesion kinase
**EndMT**	Endothelial-to-mesenchymal transition
**TIE2**	Recombinant TEK Tyrosine Kinase, Endothelial
**PRC1**	Polycomb repressive complex 1
**PRC2**	Polycomb repressive complex 2
**GPR81**	G protein-coupled receptor 81
**SalB**	Salvianolic acid B
